# Design and implementation of a medical student hazardous materials response team: the Medical Student HazMat Team

**DOI:** 10.1186/s12245-018-0195-6

**Published:** 2018-09-18

**Authors:** Joshua Verson, Nicholas Dyga, Nestor Agbayani, Fred Serafin, Louis Hondros

**Affiliations:** Department of Emergency Medicine, Rush University Medical Center, 1603 W. Congress Pkwy, Chicago, IL USA

**Keywords:** Simulation, Disaster relief, Terrorism, Emergency preparedness, Medical student, Education, Biochemical warfare, Decontamination, Emergency medicine, Disaster medicine

## Abstract

**Background:**

To design and implement a replicable disaster training curriculum for the first on-call medical student hazardous materials response team.

**Methods:**

Twenty-eight first-year medical students participated in a simulated citywide bioterrorism disaster drill. Students were notified of the Code Orange via email, a pager system, and group SMS text message. Twenty-five students participated in the drill, while the three remaining student leaders worked with the ED staff and HazMat Branch Director to ensure that all protocols were followed properly. Five groups of five students took turns donning HazMat gear, decontaminating three mannequins (an infant, a child, and an unconscious adult), and then safely removing the gear.

**Results:**

All modes of communication were received within 5 min, and all the students arrived at the ED within 20 min. The decontamination was determined to be sufficient by the team leader, Emergency Department staff, and HazMat Branch Director and was completed approximately 10 min after the entrance to the decontamination chamber.

**Conclusions:**

Current US medical school curricula lack emergency preparedness training in response to potential terrorist attacks and hazardous material exposures. Our program, while still in its early workings, not only allows students to develop critical knowledge and practical skills but also provides a unique opportunity to leverage much-needed manpower and resources during emergency situations.

## Background

In response to the increasing threat of terrorism in the USA, the Association of American Medical Colleges (AAMC) called for better coordination between medicine and public health, as well as the incorporation of disaster education into medical education curricula [1]. Health care experts found this to be highly relevant, and have consistently shown support for the proposed addition of disaster education; furthermore, they have supported increasing the role of medical students in disaster relief efforts [2, 3]. Moreover, polls have shown that the public often turns to their health care providers for guidance in cases of public health emergencies [1, 4, 5]. Even though the US terrorism index is at its highest in the past decade, the majority of medical schools in the USA still lack organized disaster medicine education, and few programs worldwide involve medical students in emergency response teams [6].

The number of host national and international health care providers trained to respond to disaster situations is very limited and can be easily overwhelmed [7]. Thus, medical student training to respond to these unfortunate scenarios should be of the upmost priority. Their involvement can be used to provide much-needed human resources, and previous studies have shown that early involvement is essential in building a strong foundation and adopting a life-long passion for the topic [1, 2]. In response, our team designed and implemented a replicable disaster-training curriculum for the development of the hospital’s first medical student hazardous materials response team. We understand that available resources can vary greatly at different institutions, but we believe that the most important aspects of this training process are quite generalizable and that the processes detailed here can serve as a structured guide and resource for other institutions to implement similar programs.

## Methods

### Medical student recruitment

In January 2016, first-year medical students were recruited to take part in the medical school’s first Medical Student HazMat Team via email and a general information meeting. Students who expressed interest met with the co-founders of the team in which they were informed about the objective and responsibilities of the HazMat team. The objective discussed was the establishment of an on-call hazardous materials response team that operates as an independent arm alongside other health care professionals in response to hazardous materials incidents or bioterrorism attacks. Responsibilities include participation in the training process, willingness to be part of an on-call team, and readiness to respond at any time. In order to maintain competency and to simulate the spontaneity of an actual terrorist attack, students are also expected to participate in drills every 6 to 9 months, with approximately 3 weeks notice. In the event of a hazardous materials incident, the team’s primary job is the triaging and decontamination of patients in the Emergency Department (ED) ambulance bay, which doubles as a decontamination area.

### Didactic and operational training

Twenty-eight medical students agreed to participate. The training process included 4 h of didactic training, 4 h of operational training, and multiple simulations. Each phase of the training was led by disaster-trained health care professionals. The Director of Emergency Management led the didactic training and taught the students about the different types of disasters (including industrial accidents, terrorist attacks, bombings, and biochemical warfare) as well as the basic triaging strategies in the field and within the ambulance bay. The operational training was completed in the ambulance bay. Students learned how to properly don (put on) the personal protective equipment (PPE), properly decontaminate and handle patients, and safely doff (remove) the PPEs to avoid contaminating themselves. Due to the gaps in the existing literature regarding the training of medical students, we utilized the same training manual that the official University Hospital emergency response team uses for their training.

**Fig. 1:**
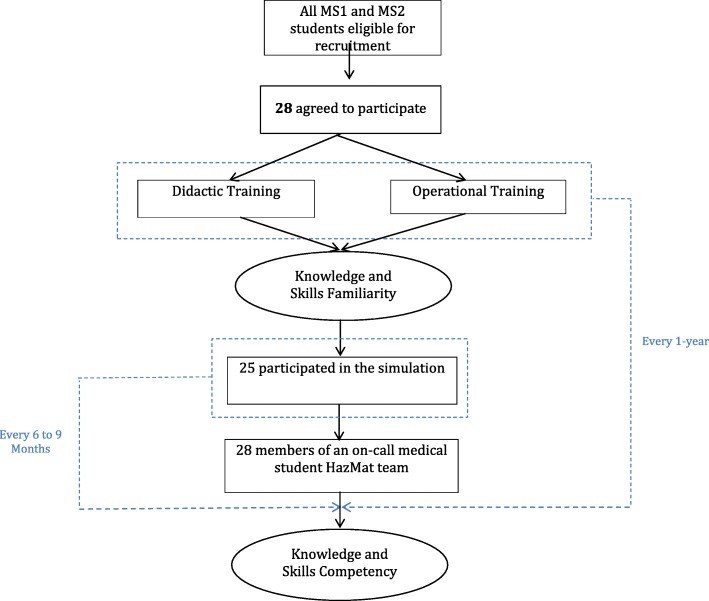
Training timeline. Timeline for training medical students in HazMat response along with assessing knowledge and skills competency during simulation

The operational training was taught by the Director of Emergency Management along with the team leaders and involved the use of mannequins that were triaged into predetermined zones designated inside the ambulance bay. Students donned in level 2 PPE began the decontamination process by first bringing the victims inside, placing them in a supine position on a conveyer belt, and then stripping off the contaminated clothing with trauma shears. The victims were then passed down the conveyer belt to get showered with high-pressure hoses and scrubbed down. Shaving cream was applied to the mannequins in various areas of their bodies to mimic the unpredictability of contamination. After the team members completed their decontamination of the simulated patient, the trainers provided feedback and pointed out any errors that may have occurred including leftover contaminants, inefficient decontamination methods, or dangerous manipulation of victims (Fig. 1).

## Results

In May 2016, the Medical Student HazMat Team joined our host academic hospital in participating in a citywide drill that simulated a large scale aerosolized release of *Bacillus anthracis*. The exercise was designed to address multiple Hospital Preparedness Program (HPP) capabilities and evaluate improvements based on information learned during 2015 hospital response efforts. The objectives focused on optimizing strategies in managing massive medical needs, coordination between emergency operations at different hospitals, information sharing, responder health and safety, and volunteer management. This citywide drill was used as an opportunity to run a small-scale Hazardous Materials Response Team (HMRT) decontamination simulation in the ED with our recently trained first-year and second-year medical students.

With 28 medical students present at the drill in May, 25 of the students performed the duties required by the simulation, while the three team leaders led the separate sections of the exercise including donning and doffing of the equipment, proper decontamination of the victims, and effective collaboration with the Emergency Department staff. The leaders worked alongside the HazMat Branch Director to ensure that all protocols were followed precisely. Due to the limited time available to initiate and complete the drill, and because the medical students had other various responsibilities, it was necessary to allow the students a 6-h window to receive a Code Orange notification and arrive at the ED for the simulated Code Orange.

Participating students were warned that they would be called in sometime between 0900 and 1500 and the day of the drill. At 0930, the team leader utilized multiple communication modalities to notify the students via email, group SMS text message, and a pager system that a Code Orange had been initiated. All modes of communication were confirmed to have been received within 5 min of sending the message, and all students arrived in the Emergency Department within 20 min of the initial notification. The team leader was informed beforehand by a Code Orange page and was waiting at the decontamination station. Other communication modalities that were considered but not used include phone trees, host hospital’s internal alert system, and social media outlets. Once all the students arrived, the personal protective equipment had to be transported from the storage closet to the staging area.

Students were broken up into five groups of five at the time of the drill, and each group had the opportunity to fully don the equipment, run through the decontamination simulation, and then safely doff the equipment. A buddy system was used so that each student had help with the proper donning and doffing of equipment. Each group then worked as a single unit to decontaminate three dummy patients: an infant, a child, and an unconscious adult. The dummy patients were dressed in hospital gowns, so the students had to first remove them with trauma shears before beginning the decontamination. Shaving cream and toothpaste were used to represent the hazardous material exposure and were distributed throughout the body including on the sites that are difficult to decontaminate such as behind the ears, behind the knees, and the axilla. Each team member rotated through the various roles with each dummy patient: cutting off the patient’s clothing, scrubbing the patient, flipping and handling the patient to clean the difficult sites of exposure, rinsing and drying, and preparing the patient for transport into the cold zone. Students had a difficulty in properly flipping the mannequins safely according to the training protocol, especially the adult mannequins that were larger in size.

**Fig. 2:**
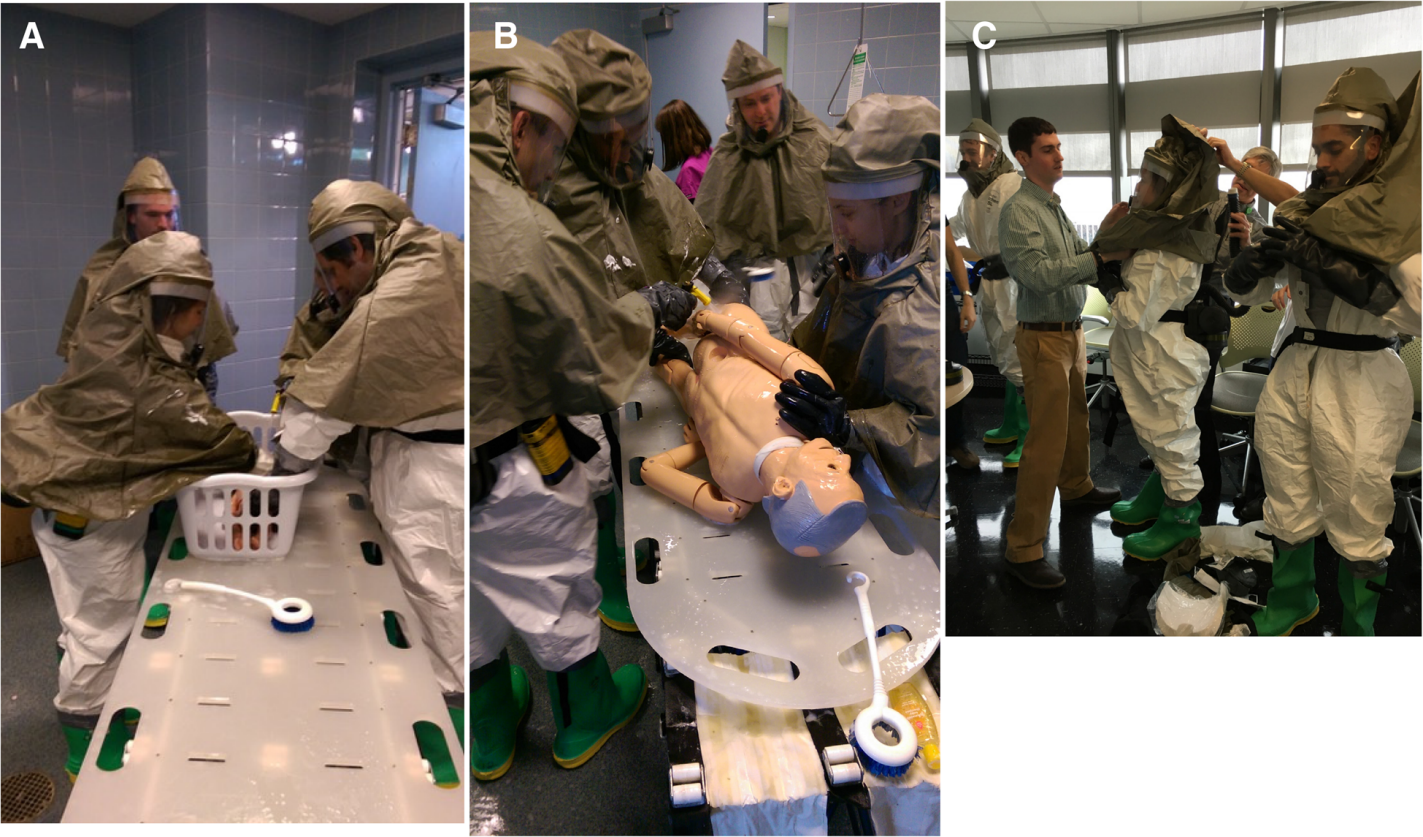
**a** Decontamination of an infant mannequin. Medical students are shown here to be decontaminating infant patients with smaller hoses and soft scrub brushes. The infant is placed in a basket and carefully cleaned. The decontamination occurs on a conveyer belt where medical students down the line have varying responsibilities. **b** Decontamination of an adult mannequin. Medical students are shown here to be decontaminating an adult mannequin with high-pressure hoses while practicing proper turning of a patient on a backboard. **c** Donning personal protective equipment. Medical students are shown here assisting each other in the donning and doffing of HazMat gear. They are working in pairs to assure that all of the equipment is secure and functioning properly

At the end of the drill, each student’s mannequin was assessed for satisfactory decontamination. From start to finish, the five groups of students were able to fully decontaminate the mannequins in an average time of approximately 10 min (Fig. 2). In a true decontamination scenario, it is more efficient to assign each team member to a single role, which allows for safe doffing of the equipment after completing his or her task. But in this drill, the rotation allowed every team member to experience each task while maneuvering around in the PPE suits. Spending an extended period of time exerting oneself while inside the suit can put the team members at risk of feeling dizzy, nauseous, and overheated, so we recommend limiting the time inside the suit to no more than 45 min. This is not a firm limit, however, and can be adjusted based on the weather and level of exertion required in the response to a given hazardous materials event. Additionally, we recommend monitoring the vital signs of the team members before donning and after doffing the suits. It is also continuously emphasized during training and simulations that team members should immediately remove themselves from a decontamination situation, whether real or simulated, if they do not feel well and are at risk of compromising the safety of themselves, their teammates, or the patients. It is also important for team members to monitor each other to monitor for any signs of fatigue.

## Discussion

Many countries have invested in various research projects that aim to improve the efficacy of disaster management [8–12]. However, despite the increasing threat of terrorism, training for this type of threat and other disasters has been focused on health care professionals and has neglected to include medical students [8, 13]. There have been multiple papers describing the importance of training and educating healthcare staff at all levels. Including a paper in the BMC Medical Education journal in which “a competency-based approach with specific measurable objectives was proposed by a national expert panel for specific levels of disaster training” [14]. The article focuses solely on theoretical objectives and training exercises based on literature searches to identify courses that already exist. To our knowledge, this pilot program is one of the first to detail a replicable disaster training curriculum for the development of a medical student disaster response team, making University Medical Center one of the first medical schools in the nation to have an on-call Medical Student HazMat Team.

One of the most effective ways to practice, troubleshoot, and improve an emergency preparedness plan or protocol is through hospital-wide drills with various simulated incidents and patients [7]. Hospitals and providers that participate in more exercises tend to perform better on objective evaluation tools that have been developed specifically to evaluate the effectiveness of emergency preparedness protocols [13]. The curriculum presented here incorporates a simulation drill following didactic and operational training that is appropriate for medical students at any level of training. We believe that this structured approach is readily adaptable and can serve as a guide and resource for others.

Our experiences with training sessions and simulations have allowed us to discover three main areas for HMRT improvement: (1) equipment access, content, and organization; (2) practical skills and decontamination procedures; and (3) communication.

### Equipment access, content, and organization

Durin5g the collection of the PPE, the medical students did not have the clearance to access the storage unit and were forced to interrupt an ED staff member to gain access. This cost the teams about 5 min and prevented another health care provider from performing his or her duties. Thus, it is important that the members of the Medical Student HazMat Team have the appropriate security clearance needed to access the necessary equipment and locations in the hospital. We also suggest a periodic equipment inspection by the team leaders to ensure that the equipment is always properly labeled and stored in an organized fashion. This will also reinforce their familiarity with the equipment.

In an actual Code Orange event that requires mass decontamination procedures, the staging area would be outside the ambulance bay while the decontamination area would be set up using the entire ambulance bay with floor to ceiling dividers splitting it up into three sections. One section would be for men, one for women, and one for families. There are connections in the ceiling for hooking hoses up to the water supply for decontamination. At the time of this drill, we were able to use the ceiling hoses in the ambulance bay but we did not have the dividers. We were able to decontaminate two victims at a time with two separate teams utilizing two separate conveyer belts.

### Practical skills and decontamination procedures

During the decontamination portion of the simulation, the teams struggled with safely handling unconscious or non-ambulatory patients. Specifically, they struggled with the transportation and repositioning of unconscious patients from supine to prone. All of the team members were first- and second-year medical students, most of whom had not yet received proper training for transporting and maneuvering patients with possible spinal cord injuries. Thus, it is critical to incorporate these skills into the operational training so that team members can repeatedly practice safe patient handling while wearing the PPE suits. Other key skills that we believe should be addressed include handling of the hospital stretcher, basic life support (BLS), or advanced cardiac life support (ACLS).

### Communication

The PPE suits made it very difficult for the medical students to communicate with one another. The suits muffled their voices, and the powered purifying respirator (PAPR) units created a steady hum that blocked out much of the surrounding noise. In addition, background noise from the hoses, sirens, patients, and other personnel all combined to make communication even more difficult. High-performance radio transmitters were utilized for every team member, but for some team members, they were difficult to use because they need at least one hand free to press the button that allows them to talk over the channel. This should improve with practice as team members become more comfortable using radio communication. It is essential that a team leader keeps the communication organized and in check, so as to allow all team members to be able to communicate clearly and effectively. When speaking through the radios in turn rather than all at once, communication between the medical students and with other integral members of the hospital personnel will allow for safer and more efficient decontamination. In light of recent threats of hacking of hospital systems, it is integral that these radio transmitters are encrypted to ensure patient privacy [15]. In the event of radio transmitter malfunction, it is also important to establish non-verbal signals that will allow team members to communicate basic information.

### Limitations and future direction

There are several limitations in this process that will make future objective evaluation difficult. Although the curriculum incorporates both didactic and operational training and real-world simulation, single simulations only allow for brief practice with disaster medicine concepts and hazardous materials decontamination skills. Medical school hazardous materials response team curriculums should include periodic workshops and drills that will allow the team members to continuously improve their skills. The observations provided at this stage, while proving valuable for continued improvement of our program, do not quantitatively measure the effectiveness of the training, specifically as it relates to the medical students’ knowledge, skills, and team efficiency. Our plan is to start incorporating objective data and outcome measures in future drills and training sessions so that we can better evaluate the effectiveness of our program. We will likely start by creating and distributing standardized questionnaires to the medical students to evaluate their disaster medicine knowledge and assess skills gained through the simulated drills. We will begin to develop checklists that can be used by an outside observer during a simulated drill that will allow team leaders to quantitatively evaluate each step of the decontamination process. We also plan to video record future drills, allowing the leaders and team to review all positive and negative outcomes and reflect on each drill. Finally, since we do not have any objective data, we cannot provide any evidence that the program positively affects patient outcomes, and there is no evidence that involving medical students in real disaster situations affects patient outcomes. The foundation has been built, however, and with this team in place, we believe that future objective measures will show that medical student involvement in hazardous materials response teams and disaster medicine as a whole will improve both patient outcomes and the competency of the nation’s future physicians in responding to disaster scenarios.

## Conclusions

According to the national security experts, it is not a matter of *if* a terrorist attack will take place on US soil; it is a matter of *when* [16]. However, despite this increasing threat, the majority of medical schools in the USA still lack organized disaster medicine education. In response, we designed and implemented a pilot program for the development of the first-ever on-call medical student hazardous materials response team. The program detailed here, while still in its early workings, allows students to develop critical knowledge and practical skills in the realm of hazardous materials response and provides the groundwork and opportunity for further study of the effects of incorporating medical students into various roles on disaster medicine response teams.
